# Mapping and Chemical Diversity of *Baccharis dracunculifolia* De Candole (1836) Essential Oil Accessed in Rio de Janeiro, Brazil

**DOI:** 10.3390/plants14223443

**Published:** 2025-11-11

**Authors:** Durval Reis Mariano-Junior, Diego da Paixão Alves, Camila da Silva Barbosa Pereira, Rosana Santos Cavalcante, Luisa Bule Reichenbach, Maria Eduarda Pereira Ribeiro, Igor Sampaio Fontes, Douglas Figueredo dos Reis Pinheiro, Mariana Emerick Silva, Lidiane Barbosa Pedro, André Marques dos Santos, Pedro Correa Damasceno Junior, Marco Andre Alves de Souza

**Affiliations:** 1 Programa de Pós-Graduação em Química, Instituto de Química, Universidade Federal Rural do Rio de Janeiro, Rio de Janeiro 23.897-000, Brazil; durvalmariano@gmail.com (D.R.M.-J.); cavalcante.rosana@gmail.com (R.S.C.); 2 Programa de Pós-Graduação em Ciências Ambientais e Florestais, Instituto de Florestas, Universidade Federal Rural do Rio de Janeiro 23.897-000, Rio de Janeiro, Brazil; diegopaixao@ufrrj.br (D.d.P.A.); camilasilvabarbosa28@gmail.com (C.d.S.B.P.); 3 Laboratório de Plantas Aromáticas e Medicinais, Universidade Federal Rural do Rio de Janeiro, Rio de Janeiro 23.897-000, Brazil; luisareich2004@gmail.com (L.B.R.); eduardalany@gmail.com (M.E.P.R.); igor.sampaiof73@gmail.com (I.S.F.); douglasreis@ufrrj.br (D.F.d.R.P.); silva.marianaemerick@gmail.com (M.E.S.); lidianebpedro@gmail.com (L.B.P.); 4 Departamento de Bioquímica, Instituto de Química, Universidade Federal Rural do Rio de Janeiro, Rio de Janeiro 23.897-000, Brazil; amarques@ufrrj.br; 5 Departamento de Agrotecnologia e Sustentabilidade, Instituto de Agronomia, Universidade Federal Rural do Rio de Janeiro, Rio de Janeiro 23.897-000, Brazil; damascenojunior2009@gmail.com

**Keywords:** chemodiversity, Asteraceae, volatiles oils, chemotyping, terpenes

## Abstract

Brazil is recognized for its rich biodiversity, including aromatic species of economic importance, among which *Baccharis dracunculifolia* De Candole (1836) stands out. The essential oil distilled from this species exhibits biological and therapeutic activities. Despite its relevance, studies addressing the chemodiversity of this species on a broad scale remain scarce. This study aimed to map and characterize the chemical and physicochemical profiles of *B. dracunculifolia* essential oils from different regions of the state of Rio de Janeiro, considering the influence of geographic factors and plant sex. Fifty georeferenced accessions of *B. dracunculifolia* were collected in 2023 and 2025, and dried leaves were subjected to hydrodistillation. The essential oils were characterized through physicochemical analyses and chemically analyzed by GC-FID and GC-MS. Essential oil yields ranged from 0.34 to 2.17%, relative density from 0.89 to 0.96 g/cm^3^, refractive index from 1.485 to 1.497 n^D^, and specific optical rotation from −12.56° to +6.80°. Sixty-two compounds were identified, predominantly oxygenated sesquiterpenes, with *E*-nerolidol (16.8–51.0%), spathulenol, bicyclogermacrene, and germacrene D as the main compounds. Multivariate analysis revealed five chemical profiles, all containing *E*-nerolidol as the major compound, indicating moderate to low chemical diversity. No significant differences were observed between the essential oils from female and male plants. However, variation in the chemical profile of the essential oil was observed as a function of year and altitude.

## 1. Introduction

The Convention on Biological Diversity (CBD) defines biological diversity as the variability among living organisms from all sources, including terrestrial, marine, and other aquatic ecosystems, as well as the ecological complexes of which they are part, emphasizing the diversity within and among species [1]. Brazil harbors immense biodiversity, encompassing six terrestrial biomes, transition zones between them, and specific environmental conditions that create regions of high biodiversity [2]. It is important to note that, similar to intraspecific polymorphism, chemodiversity also plays a major role in enhancing biodiversity within these biomes.

Among environments with high biodiversity, several aromatic and medicinal plants stand out, including *Baccharis dracunculifolia* De Candole (1836) [Asteraceae], a native Brazilian species commonly found in areas of ecological succession and pastures. It is a dioecious shrub species known as *Field rosemary* or *Broom plant* (known in Portuguese as *Vassourinha* or *Alecrim-do-campo*), widely used as a forage source for bees that produce green propolis, which contains artepillin C as its main bioactive compound of medicinal interest [3,4].

Plants of the genus *Baccharis*—for example, *B. trimera* (“carqueja”) and *B. dracunculifolia*—are traditionally used for the management of gastrointestinal and hepatic disorders and inflammatory conditions, and they also provide an important source of income for riverside, coastal, Indigenous, and quilombola communities in Brazil [5,6,7]. Moreover, this genus has attracted considerable scientific interest, since several of its species exhibit relevant biological activities, including antimicrobial, antioxidant, antiviral, antifungal, antidiabetic, antipyretic, and gastroprotective effects. Most of these biological properties have been demonstrated in extracts prepared from the aerial parts and leaves of *Baccharis* species, particularly in essential oils and hydroethanolic or ethanolic extracts. The essential oil of *B. dracunculifolia*, in particular, exhibits anti-inflammatory, antifungal, antioxidant, and acaricidal properties [8,9,10,11,12].

In summary, essential oils are plant-derived products obtained by distillation [13], composed mainly of volatile and semi-volatile organic compounds with low water solubility [14]. The essential oils market is dynamic and globalized, encompassing several industrial sectors, including the fragrance and flavor industries and, in particular, the rapidly expanding field of aromatherapy, which has significantly increased the global demand for essential oils [15]. To ensure the quality of essential oils, chemical and physicochemical characterization is recommended in accordance with international standards [16,17,18,19].

Recent research by Furlan et al. [20] has contributed to this field by employing comprehensive two-dimensional gas chromatography (GC×GC) to explore the essential-oil profile of *B. dracunculifolia* and its seasonal variability. Although that study focused on 15 individuals from a single municipality, it provides valuable insights and complements previous works that collectively highlight the metabolic richness and ecological plasticity of this species. Therefore, broader mapping and chemical characterization efforts remain essential to fully capture the diversity of *B. dracunculifolia* across its natural range and to support strategies for genetic conservation and the selection of superior genotypes for essential oil production.

Despite the existence of some studies on *B. dracunculifolia*, investigations that simultaneously address the chemical, physicochemical, and spatial dimensions of its essential oils remain scarce. In this study, 50 georeferenced individuals collected from several municipalities across the state of Rio de Janeiro were analyzed, encompassing distinct environmental and temporal conditions. This approach allowed a detailed examination of the intraspecific variability in essential oils, integrating physicochemical parameters with comprehensive chromatographic profiling and multivariate analyses to reveal chemical patterns and their regional distribution. The simultaneous consideration of plant sex and sampling period further expanded the analytical scope, offering new perspectives on the influence of biological and seasonal factors on the species’ chemical diversity.

## 2. Results

### 2.1. Collection, Yield, and Physicochemical Analysis of Essential Oils

**Figure 1:**
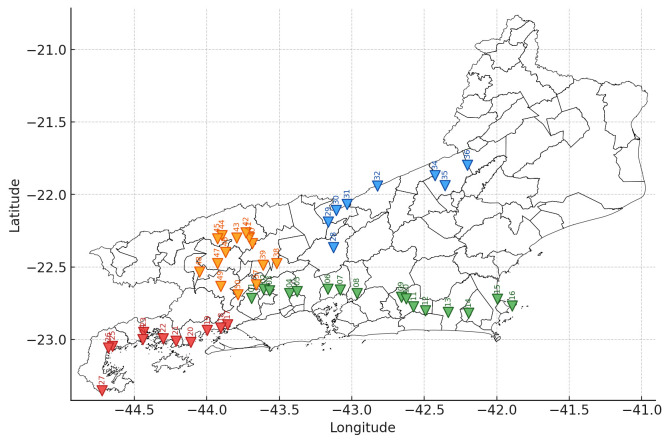
Spatial distribution of the 50 *Baccharis dracunculifolia* samples collected in the state of Rio de Janeiro. The inverted triangles indicate the geographical position of each accession, numbered from BD-P01 to BD-P50. Colors represent different collection routes: green (Metropolitana and Baixada Litorânea Regions), red (Costa Verde Region), orange (Centro-Sul and Médio Paraíba Regions), and blue (Centro-Sul and Serrana Regions).

*Baccharis dracunculifolia* plants accessed in different localities of the state of Rio de Janeiro were collected, labeled, and georeferenced (Figure 1 and Table 1). Part of the plant material was used for taxonomic identification and deposited in the herbarium, while another portion was destined for hydrodistillation. Although the collection areas are organized according to the political–administrative regions of the state of Rio de Janeiro, these divisions do not correspond to homogeneous edaphoclimatic units.

**Figure 2:**
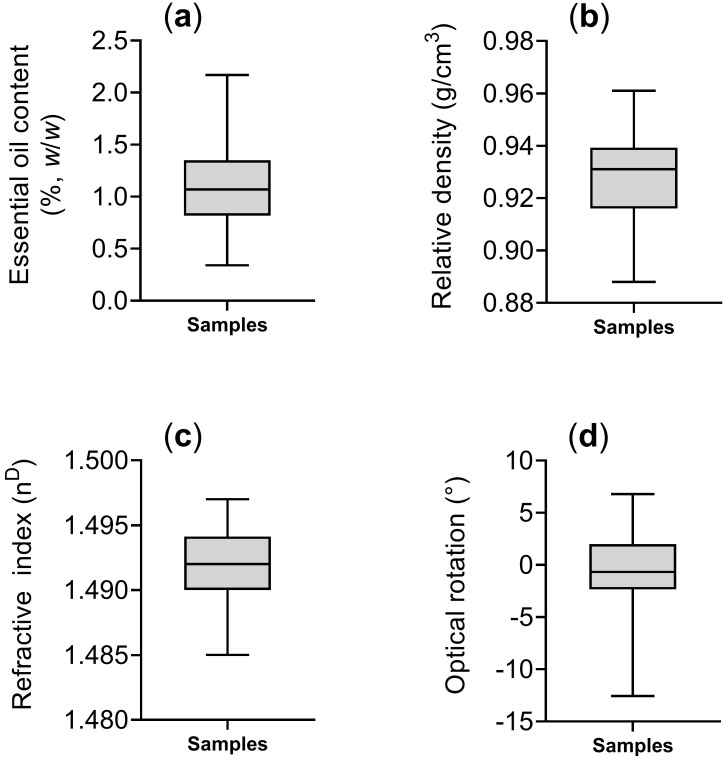
Statistical descriptions of essential oil content (**a**), relative density (**b**), refractive index (**c**), and optical rotation (**d**) in *Baccharis dracunculifolia* samples collected in Rio de Janeiro, Brazil. Boxplots show quartile distribution, with the central line representing the median.

The essential oils obtained were subjected to gravimetric analysis to determine the essential oil yield, which ranged from 0.34 to 2.17%, with a mean and standard deviation of 1.10 ± 0.38%, respectively (Figure 2a). Subsequently, the essential oils were analyzed for their physicochemical properties. The relative density ranged from 0.89 to 0.96 g/cm^3^, with a mean and standard deviation of 0.93 ± 0.02 g/cm^3^ (Figure 2b). The refractive index varied between 1.485 and 1.497, with a mean and standard deviation of 1.492 ± 0.003 n^D^ (Figure 2c). The optical rotation ranged from –12.56° to +6.80°, with predominantly negative values, and a mean and standard deviation of −0.77° ± 4.23° (Figure 2d).

**Figure 3:**
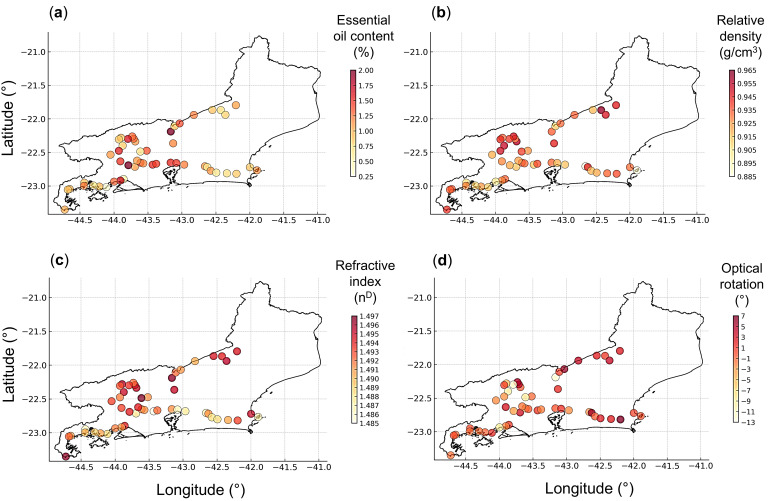
Georeferenced symbol map with a graduated color scale representing the spatial distribution and relative values of essential oil content (**a**), relative density (**b**), refractive index (**c**), and optical rotation (**d**) across *Baccharis dracunculifolia* samples collected in Rio de Janeiro, Brazil.

The variables presented in Figure 2 were georeferenced on a map of the state of Rio de Janeiro, using symbols and color gradients (Figure 3). The essential oil yield showed higher values in samples from the Metropolitana, Serrana, Centro-Sul, and Médio Paraíba Regions, specifically between longitudes −43 and −44 (Figure 3a). Higher relative density values were more frequent in the Serrana and Médio Paraíba Regions, especially above latitude –22.5 (Figure 3b). The refractive index exhibited a relatively homogeneous distribution; however, higher values were more frequently observed in the Centro-Sul, Serrana, and Médio Paraíba Regions, whereas lower values occurred predominantly in the Baixada Litorânea, Costa Verde, and Metropolitana Regions (Figure 3c). The optical rotation showed a rather homogeneous distribution; however, lower values were more frequently found in the Médio Paraíba and Centro-Sul Regions (Figure 3d).

### 2.2. Chemical Composition of Essential Oils

**Figure 4:**
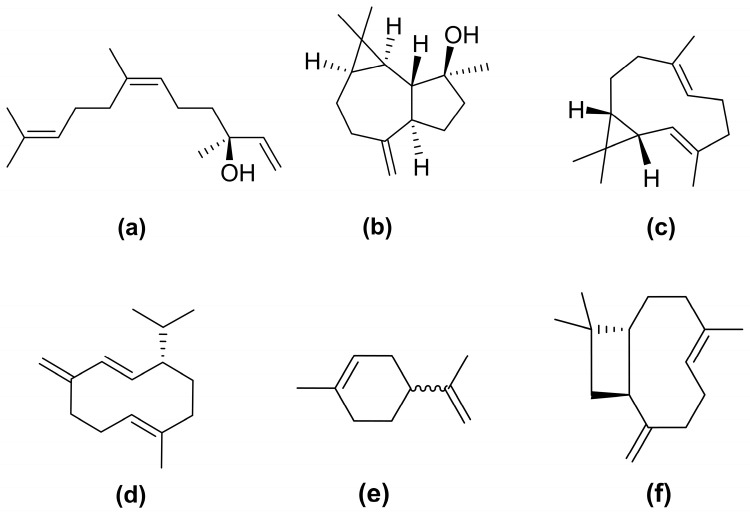
Chemical structures of the main sesquiterpenes identified in *Baccharis dracunculifolia* essential oil: *E*-nerolidol (**a**), spathulenol (**b**), bicyclogermacrene (**c**), germacrene D (**d**), limonene (**e**), and E-caryophyllene (**f**).

The chemical analysis of 50 essential oil samples of *B. dracunculifolia* resulted in the identification of 62 compounds, representing 91–100% of the total composition (Table S1). From this total, 23 compounds were selected as the most relevant for characterizing the chemical profile of the species, among which 15 showed a frequency higher than 95% (Table 2). The most abundant compound was *E*-nerolidol, detected in 100% of the essential oil samples, with contents ranging from 16.8% to 51.0% (Figure 4). Eight other compounds were also present in all samples (100% frequency): *E*-caryophyllene (1.3–10.1%), aromadendrene (0.4–3.6%), *α*-humulene (0.7–2.0%), germacrene D (2.5–15.2%), bicyclogermacrene (3.0–14.5%), δ-cadinene (1.6–5.3%), spathulenol (3.3–19.5%), and globulol (1.4–7.0%). Compounds with frequencies between 95% and 99% included *β*-pinene (0.5–7.5%), limonene (0.9–10.5%), cabreuva oxide B (0.7–3.4%), *γ*-cadinene (0.5–1.5%), and *α*-cadinol (1.4–5.1%).

**Figure 5:**
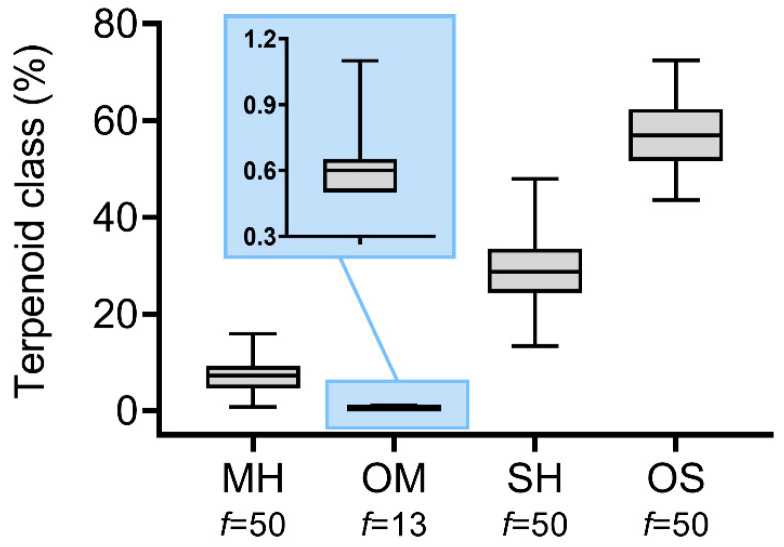
Statistical descriptions of terpenoid classes (MHs—monoterpene hydrocarbons; OMs—oxygenated monoterpenes; SHs—sesquiterpene hydrocarbons; and OSs—oxygenated sesquiterpenes) in *Baccharis dracunculifolia* essential oil samples collected in Rio de Janeiro, Brazil. Boxplots show quartile distribution, with the central line representing the median. *f*—absolute frequency of one or more compounds belonging to each terpenoid class in the essential oil samples.

The analysis of terpene classes revealed that monoterpene hydrocarbons ranged from 0.8 to 16.0%, with a mean value of 7.3%, while oxygenated monoterpenes were poorly represented, being detected in 13 samples with contents between 0.5 and 1.1% (Figure 4). Sesquiterpene hydrocarbons ranged from 12.9 to 45.9%, with a mean value of 27.8%. Oxygenated sesquiterpenes constituted the major class, varying from 46.0 to 73.7%, with a mean value of 58.8% (Figure 5).

### 2.3. Chemodiversity of Essential Oils

Diversity indices were calculated based on the chemical composition and the distribution of compounds in the essential oils of *B. dracunculifolia* obtained from the study region, resulting in the following values: Shannon (H′ = 0.849), Simpson (1−D = 0.489), Pielou (J = 0.773), and Margalef (Mg = 0.535). Taken together, these indices indicate that the chemical diversity in the study region tends to range from moderate to low.

**Figure 6:**
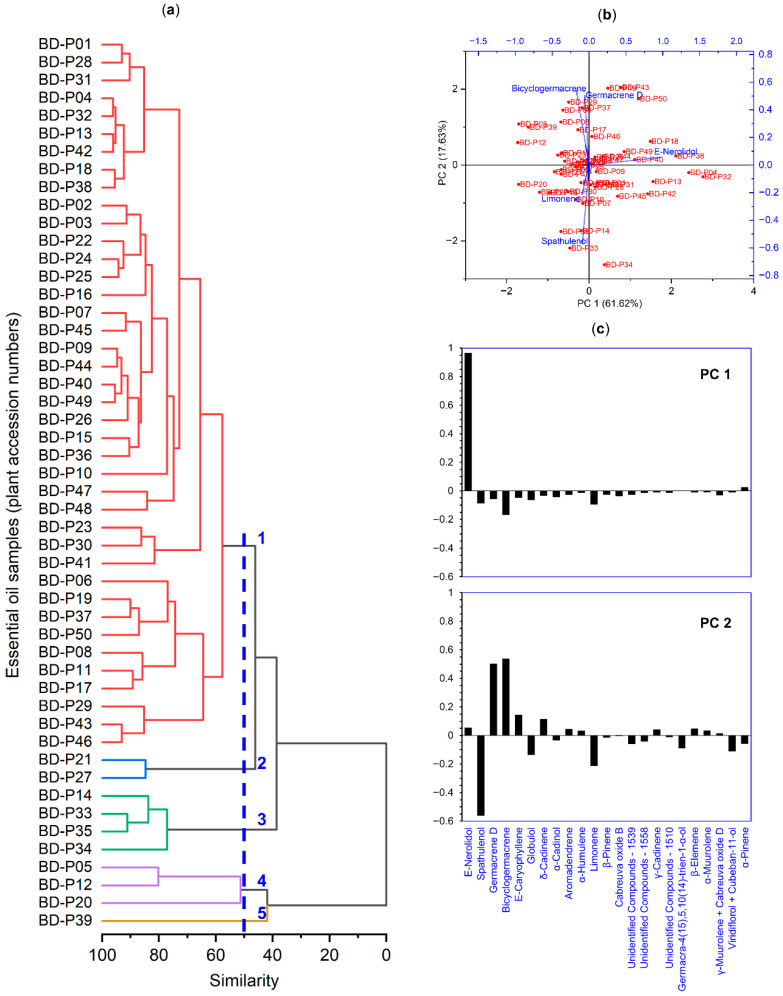
(**a**) Hierarchical cluster analysis (HCA). The dendrogram was constructed using the unweighted pair-group method with arithmetic mean (UPGMA) and the Pearson correlation coefficient estimated between the independent variables (50 essential oils of *Baccharis dracunculifolia*) and the dependent variables (23 chemical compounds). The blue dashed line represents the cutoff used to 50% similarity, and the numbers (1 to 5) indicate the groups formed by the cut in the dendrogram. (**b**) Biplot representing the principal component analysis (PCA). Red labels indicate sample dispersion (scores), and blue labels indicate the contribution of compounds to the dispersion (loadings). (**c**) Factor loadings of chemical compounds on the first two principal components (PC1 and PC2) obtained by PCA. Bars indicate the relative contribution and direction of each compound to the variance explained by each component.

Hierarchical cluster analysis grouped the 50 essential oil samples into five main clusters according to the similarity of their chemical profiles (Figure 6a). The cophenetic correlation coefficient, which indicates the quality of the fit between the phenetic and cophenetic matrices, was 0.78. Principal component analysis (PCA) explained 79.47% of the total variance, with 61.62% accounted for by the first component (PC1) and 17.85% by the second (PC2) (Figure 6b). Cluster 1, which included most of the samples, was associated with higher values of *E*-nerolidol. Cluster 3 was separated due to the higher contribution of spathulenol, whereas clusters 2, 4, and 5 showed greater influence from *E*-caryophyllene, germacrene D, and bicyclogermacrene in different proportions (Figure 6b,c).

The application of a classification key based on the dominance of one, two, or three major compounds allowed the definition of four chemotypes for the essential oils of *B. dracunculifolia* (Table 3) collected in the state of Rio de Janeiro. The *E*-nerolidol chemotype was the most representative, comprising 33 accessions in which this major compound was dominant. A second chemotype, represented by accessions BD-P33 and BD-P34, exhibited the *E*-nerolidol/spathulenol pattern, characterized by the codominance of these two compounds. The third chemotype, represented by a single accession (BD-P20), was defined by the dominance of *E*-nerolidol/spathulenol/bicyclogermacrene. In a total of 14 accessions, no single compound was dominant, corresponding to a mixed (M) or undefined chemical pattern.

**Figure 7:**
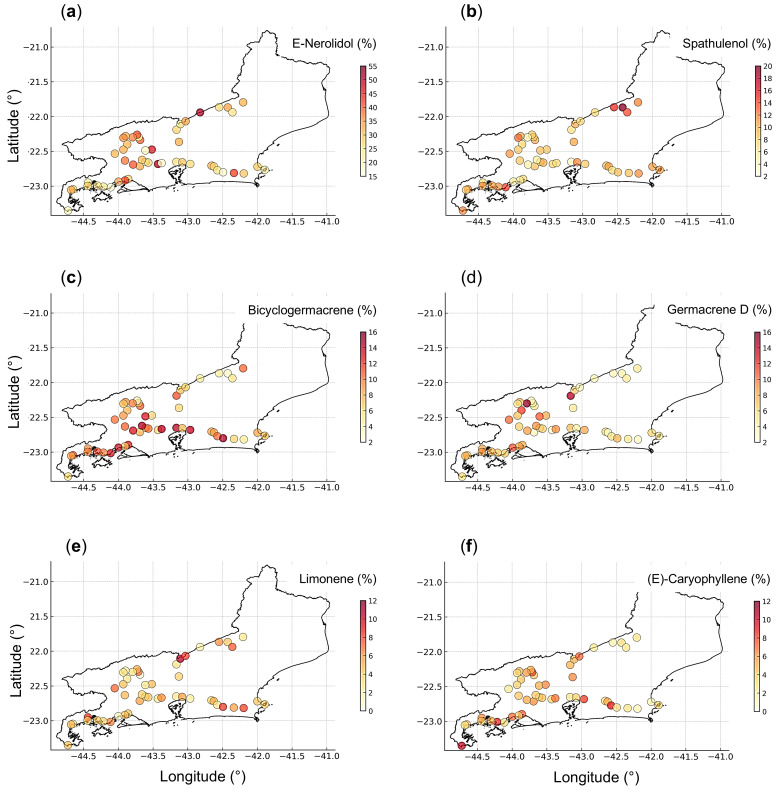
Georeferenced symbol map with a graduated color scale representing the spatial distribution and relative proportions of *E*-nerolidol (**a**), spathulenol (**b**), bicyclogermacrene (**c**), germacrene D (**d**), limonene (**e**), and *E*-caryophyllene (**f**) in the essential oil samples of *Baccharis dracunculifolia* collected in Rio de Janeiro, Brazil.

The spatial distribution of compounds revealed patterns associated with the physiographic and phytophysiognomic diversity of Rio de Janeiro State (Figure 7). Plants with higher *E*-nerolidol contents were mainly concentrated in the Metropolitana and Médio Paraíba Regions, particularly in areas of intermediate to high elevation (400–700 m), characterized by humid climates and milder temperatures. Spathulenol was more abundant in samples from the Serrana Region, consistent with montane forest environments. In contrast, bicyclogermacrene showed higher levels in the Metropolitana and Costa Verde Regions, both characterized by lower elevations and warmer coastal climates. Lower germacrene D levels were predominantly observed in the Serrana Region, which includes higher-elevation sites, and in the Baixadas Litorâneas, where soils are sandy, nutrient-poor, and located near the coast. Limonene exhibited a relatively homogeneous spatial distribution across the sampling sites, whereas higher proportions of *E*-caryophyllene were mainly detected in the Costa Verde Region, where steep slopes and higher humidity prevail.

Given the distinct landscape features of the state of Rio de Janeiro and the variations related to plant sex and harvest time, it became essential to investigate the influence of these factors on the chemical profile of *B. dracunculifolia* essential oils. The PERMANOVA analysis (*F* = 2.296; *R*^2^ = 0.092; *p* = 0.016) revealed significant differences in essential oil composition among the three evaluated altitudinal ranges (Table 4). Pairwise comparisons indicated that the most pronounced variations occurred between the lowest (0–100 m) and highest (400–700 m) elevations (*p* = 0.011), while the intermediate group did not differ significantly from either extreme. These results indicate that altitude, rather than the political–administrative regional division, exerts a stronger influence on the chemical variability of *B. dracunculifolia*.

**Figure 8:**
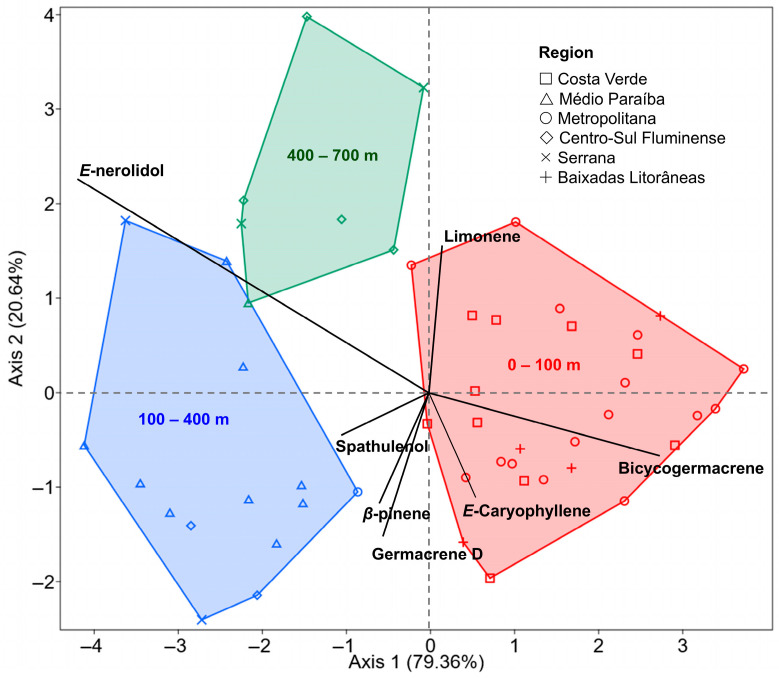
Linear discriminant analysis (LDA) of *Baccharis dracunculifolia* essential oil profile across three altitudinal ranges (0–100, 100–400, and 400–700 m). The first two discriminant functions explained 79.36% and 20.64% of the total variance, respectively. Symbols represent samples grouped by political–administrative regions of Rio de Janeiro State, while colored polygons delimit the altitudinal groups. Vectors indicate the main compounds contributing to group separation.

The discriminant analysis confirmed this pattern, showing clear segregation among the altitudinal groups, with the first two discriminant functions explaining 79.4% and 20.6% of the total variance, respectively (Figure 8). *E*-nerolidol was more abundant at higher elevations, suggesting adaptive responses to environmental stress factors such as increased radiation and lower temperatures, whereas bicyclogermacrene predominated at lower elevations, typically associated with warmer conditions and proximity to the coast.

The comparison between the two collection periods (2023 and 2025) revealed a statistically significant difference in the multivariate chemical profiles (*F* = 3.531, *R*^2^ = 0.068, *p* = 0.005) (Table 4). This result indicates that the collection period accounted for approximately 7% of the total variance, suggesting a measurable moderate temporal effect on the essential oil composition of *B. dracunculifolia.* Such variation may be associated with seasonal or phenological differences between the sampling periods, which correspond to late spring–early summer (2023) and mid-summer (2025).

Considering that the study included individuals of different sexes, a multivariate approach (PERMANOVA) was applied to assess whether this biological factor could influence the overall chemical composition of *B. dracunculifolia* essential oils (Table 4). Comparisons of the chemical profiles revealed no statistically significant differences between female, male, and individuals without sex reported (*F* = 1.826, *R*^2^ = 0.059, *p* = 0.065). When only individuals with defined sex were compared (female × male), the result remained non-significant (*F* = 1.516, *R*^2^ = 0.072, *p* = 0.199), indicating that plant sex did not exert a detectable influence on the multivariate chemical profile (Table 4).

**Figure 9:**
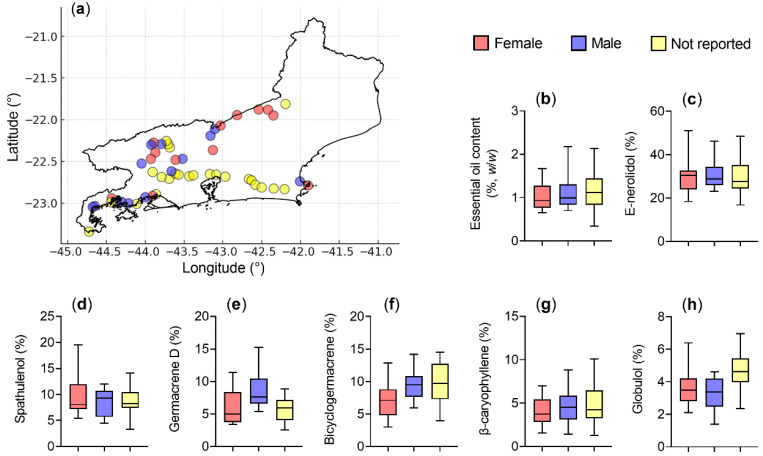
Geographic distribution and chemical variation in *Baccharis dracunculifolia* according to plant sex. (**a**) Occurrence map of samples collected in the state of Rio de Janeiro, indicating female plants (red), male plants (blue), and plants with no sex reported (yellow). Boxplots representing the variation in essential oil content (**b**) and in the main chemical compounds: *E*-nerolidol (**c**), spathulenol (**d**), germacrene D (**e**), bicyclogermacrene (**f**), *E*-caryophyllene (**g**), and globulol (**h**). The central line represents the median, boxes indicate quartiles, and whiskers show minimum and maximum values.

To evaluate the possible influence of plant sex on specific chemical constituents, the main components of the essential oils were analyzed individually (Table 5). The results, presented as boxplots (Figure 9), showed no statistically significant differences between female and male plants for any evaluated parameter, including essential oil yield and the proportions of *E*-nerolidol (*F* = 0.002; *p* = 0.969), spathulenol (*F* = 0.944; *p* = 0.341), germacrene D (*F* = 2.505; *p* = 0.126), bicyclogermacrene (*F* = 3.003; *p* = 0.096), *E*-caryophyllene (*F* = 1.556; *p* = 0.499), and globulol (*F* = 0.871; *p* = 0.360). One-way ANOVA confirmed the absence of significant differences between sexes, consistent with the PERMANOVA results obtained for the complete multivariate chemical profile. Together, these analyses indicate that plant sex had no measurable effect on either individual compound abundance or overall essential oil composition.

## 3. Discussion

The results obtained in this study contribute to understanding the genetic structure of *Baccharis dracunculifolia* based on the chemical composition and physicochemical characteristics (relative density, refractive index, and optical rotation) of 50 essential oils from plants collected in the state of Rio de Janeiro. The observed variation in essential oil content (0.34–2.17%) demonstrates the plasticity of this trait in the species. However, the relative influence of genetic, edaphoclimatic, or ecological factors on this variation could not be determined from the present data, as these factors were not experimentally isolated. Therefore, it was not possible to establish which of them—or whether their combined action—is responsible for the observed diversity. Additionally, multivariate analyses were used to assess whether biological (sex), temporal (collection period) and geographic (altitude) factors could influence the overall chemical profile.

Methodological differences among studies often hinder direct comparisons of essential oil yields. Factors such as distillation time, apparatus type, steam flow rate, and the physiological condition of plant material vary considerably. Nevertheless, an analysis of 11 scientific articles revealed yields ranging from 0.11% to 1.8% [21,22,23,24,25,26,27,28,29,30,31], which corroborates the values obtained in the present study.

Few studies have reported the physicochemical parameters of *B. dracunculifolia* essential oils, making consistent comparisons difficult. Fabiane et al. [21], however, analyzed oil from specimens collected in Paraná and reported a relative density of 0.915 g/cm^3^, a refractive index of 1.4593, an optical rotation of +1.99°, and a yield of 1.54%. These values are aligned with those observed in the present study (Figure 2). In this study, five samples (BD-P19, BD-P29, BD-P39, BD-P43, and BD-P46) showed optical rotation values lower than the average (ranging from −9.15° to −12.56°), thus being considered potential outliers. This variation may be attributed to the different proportion of chiral compounds with enantiomeric configurations. However, no chiral GC×GC analyses were performed to confirm the origin of this optical behavior.

The essential oils of *B. dracunculifolia* were characterized by a restricted set of compounds accounting for more than 85% of the total composition, with frequencies above 74% among individuals, indicating a recurrent and stable chemical profile. Statistical analyses (Table 4) revealed a moderate effect of the collection year (*R*^2^ = 0.068) and altitude (*R*^2^ = 0.092), suggesting that while the qualitative chemical profile remains largely stable, slight quantitative fluctuations among years contribute modestly to overall variance. This subject will be discussed in greater depth later in the text.

As observed in this work, this pattern reflects a moderate-to-low level of chemical diversity, distributed across five main profiles: one predominantly rich in *E*-nerolidol and four others mainly differentiated by relative proportions of spathulenol, germacrene *D*, bicyclogermacrene, and *E*-caryophyllene (Figure 6 and Table 3). The calculated diversity indices indicate a high frequency of major compounds accompanied by moderate evenness among the remaining constituents. Such chemical organization has been reported for other aromatic and medicinal species, reinforcing previous observations for *B. dracunculifolia* [32,33,34].

*E*-Nerolidol is consistently reported as the major compound in *B. dracunculifolia* essential oils, with concentrations ranging from 3.6% to 33.5% [24,26], and is considered a specific chemical marker of the species [4]. Nevertheless, its absence in some populations suggests the occurrence of distinct chemotypes [3,35]. Other sesquiterpenes, such as spathulenol (1.6–27.4%), bicyclogermacrene (1.2–19.2%), and germacrene D (0.7–21.5%), are also frequently detected [21,22,23,31,36].

Recent studies reinforce the influence of seasonal and climatic factors on the volatile composition of *B. dracunculifolia*. Furlan et al. [20] observed that even within a single Cerrado population, oil yield and composition varied moderately between seasons, with an increase in oxygenated sesquiterpenes such as *E*-nerolidol during the rainy period. Similarly, Tomazzoli et al. [37] reported higher yields in summer, associated with changes in the proportion of monoterpenes and sesquiterpenes. These findings indicate that although seasonal fluctuations are evident, they do not substantially alter the predominant chemical profile of the species, consistent with the moderate temporal (collection period) and geographic (altitude) chemical profile variation observed in the present study (Table 4). Together, these results suggest that environmental factors can modulate the relative abundance of specific compounds without changing greatly the overall chemical profile of the species.

The chemical diversity of *B. dracunculifolia* has generally been reported as low, even among geographically distant populations. Rigotti et al. [38] analyzed natural Cerrado populations and identified three main groups with very similar profiles dominated by *E*-nerolidol and, to a lesser extent, spathulenol, also suggesting chemical profile stability under different environmental conditions. Complementarily, Monteiro et al. [36] found regional differences among samples from the Central–West, Southeast, and South, with higher *E*-nerolidol and spathulenol contents in Central–West populations. Conversely, Sousa et al. [39] demonstrated that cultivation under controlled conditions reduces intraspecific variability, maintaining homogeneous chemical profiles. Altogether, these findings indicate that the chemical composition of *B. dracunculifolia* reflects the interaction between genetic and environmental factors and cultivation practices, with a moderated diversity of chemical profile, mostly restricted to three or four compounds with a high frequency across different populations.

From an applied perspective, the chemical profiles observed in this study exhibit pharmacological relevance [40]. The predominant *E*-nerolidol chemotype has been widely reported for its anticholinesterase, antioxidant, antinociceptive, anti-inflammatory, and anxiolytic properties [41,42]. Likewise, spathulenol displays antioxidant, anti-inflammatory, antiproliferative, and antimycobacterial activities [43,44], while bicyclogermacrene has been associated with the anti-inflammatory potential of *Piper gaudichaudianum* and *P. mikanianum* oils [45]. Furthermore, germacrene *D* plays ecological roles in plant–insect interactions [46] and has demonstrated insecticidal potential [47].

The occurrence of multiple chemical profiles within a species indicates the presence of essential oils with distinct sensory and biological characteristics, offering opportunities for the selection of specific genetic resources for different economic applications. This highlights the importance of conserving intraspecific chemical diversity through active germplasm collections—a strategy already adopted by several research centers [48,49,50,51]. The moderate yet measurable temporal variation observed in this study reinforces the value of maintaining this diversity under controlled agronomic conditions.

For example, in a germplasm collection maintained at UFRRJ comprising 42 genotypes of *Lippia alba*, two accessions—UFRRJ ECB 0003/008 (carvone/limonene) and UFRRJ ECB 028 (linalool)—showed promising results for controlling the pest insect *Callosobruchus maculatus* [52]. Similarly, among five chemotypes of *Varronia curassavica*, the accession ESB45, characterized by the (2E,6Z)-farnesol/(2E,6E)-methyl farnesoate chemotype, exhibited the highest activity against *Trypanosoma cruzi* [53].

The predominance of *E*-nerolidol, spathulenol, or bicyclogermacrene in different regions supports the influence of local environmental conditions on metabolic modulation [54]. In addition to altitude differences, explaining 9.2% of chemical variance, a moderate temporal (2023 to 2025 collections) component also was detected, explaining 6.8% of total chemical variance, both consistent with seasonal or phenological effects between sampling periods (Table 4 and Figure 8). The phenotypic variability observed in *B. dracunculifolia* essential oils, considering the geographic distribution of accessions across regions with distinct edaphoclimatic characteristics (Figure 1 and Figure 8), reflects a genotype–environment interaction that warrants further investigation to elucidate the contribution of each factor, as discussed by Duarte Junior et al. [55].

This study demonstrated no significant sex-related differences in essential oil yield, physicochemical parameters, or proportions of major compounds (Figure 9; Table 4 and Table 5). Consistent with these findings, Armstrong et al. [4] reported that, despite minor quantitative variations between male and female plants, both sexes share highly similar volatile profiles.

Overall, these findings underscore the strategic importance of investing in research, conserving genetic resources through ex situ collections, and valuing biodiversity in all its complexity as potential sources of innovation for the development of raw materials and bio-based technologies in sectors such as agribusiness, health, and biotechnology.

## 4. Materials and Methods

### 4.1. Research Authorization

This study was registered in the National System for the Management of Genetic Heritage and Associated Traditional Knowledge (Sistema Nacional de Gestão do Patrimônio Genético e do Conhecimento Tradicional Associado—SisGen, registration code A762930) and obtained the necessary authorizations for field material collection from the Rio de Janeiro State Environmental Institute (Instituto Estadual do Meio Ambiente do Rio de Janeiro—INEA, authorization No. 061/2022) and from the Chico Mendes Institute for Biodiversity Conservation (Instituto Chico Mendes de Conservação da Biodiversidade—ICMBio, authorization code 87594-2).

### 4.2. Plant Prospecting and Collection

The collection area was initially selected based on a preliminary analysis of terrain and vegetation carried out using a geovisualization program based on satellite imagery and geographic data [56]. The sampling method consisted of the random selection of individuals of *B. dracunculifolia* DC. (Asteraceae) within a predefined area, ensuring a minimum distance of 3 km between native specimens. The in situ collections were conducted in 28 municipalities across six regions of the state of Rio de Janeiro (Brazil). The first collection included 27 plants sampled in the Costa Verde, Metropolitan, and Coastal Lowlands regions between 5 October and 13 December 2023, while the second included 23 individuals collected in the Serrana, South–Central Fluminense, Middle Paraíba, and Metropolitan regions between 15 January and 5 February 2025, totaling 50 samples (Table 1 and Figure 1).

The Metropolitan Region comprises lowlands and gentle hills with elevations ranging from 0 to 100 m above sea level, a hot and humid tropical climate, and remnants of Atlantic Forest on the slopes. The Coastal Lowlands (Baixadas Litorâneas) are near sea level (0–80 m), with a humid climate under maritime influence and restinga vegetation interspersed with coastal Atlantic Forest. The Costa Verde (Green Coast) corresponds to the coastal sector of the Serra do Mar, where elevations vary from sea level to about 100 m, with steep terrain and dense humid forests. The South–Central Fluminense Region features undulating relief and elevations between 170 and 340 m, with a tropical climate of moderate altitude and secondary Atlantic Forest vegetation. The Middle Paraíba Valley includes valleys and gentle hills along the Paraíba do Sul River basin, with elevations between 370 and 690 m and riparian forests under a humid tropical climate. Finally, the Serrana Region (Mountain Region) has mountainous relief, higher elevations ranging from 360 to 480 m, a mild and humid climate, and montane rainforests associated with slope-drainage areas (Table 1).

The collected material (branches containing leaves and inflorescences) was in either vegetative or reproductive stage and was identified using the code “BD-P” followed by a sequential number from 1 to 50, corresponding to each plant sampled in the field. All specimens were georeferenced (Table 1). The branches containing leaves, flowers, and/or fruits were sent to the Herbarium of the Universidade Federal Rural do Rio de Janeiro (UFRRJ) for the preparation of voucher specimens and assignment of corresponding accession numbers (Table 1). The plant materials intended for essential oil extraction were subjected to a drying process and stored according to the literature [53] until the time of essential oil extraction.

### 4.3. Essential Oil Distillation

Essential oil extraction was carried out at the Laboratory of Aromatic and Medicinal Plants (Laboratório de Plantas Aromáticas e Medicinais) of the Federal Rural University of Rio de Janeiro (Universidade Federal Rural do Rio de Janeiro—UFRRJ), located in the municipality of Seropédica, Rio de Janeiro, Brazil. The essential oils were obtained by hydrodistillation using a Clevenger-type apparatus. For each extraction, between 70 and 280 g of fine branches, leaves, and flowers were used, depending on the availability of each sample. The plant material was previously dried, ground, and placed in a 5 L round-bottom flask containing 3 L of distilled water. The distillation process was conducted for 4 h at a steam flow rate of 3 mL/min, a condition determined from a distillation kinetics study (data not yet published). Complete separation and recovery of the essential oil, as well as determination of yield (%, *w*/*w*, dry weight basis), were performed according to the literature [53].

### 4.4. Physicochemical Analyses

The refractive index of the essential oils was determined according to ISO 280:1998 [17], using 20 µL of each essential oil sample at 26 ± 0.1 °C, with an Abbe-type bench refractometer (model Abbe FOR-1000S). The specific optical rotation was measured according to ISO 592:1998 [18], using 1.6 mL of a 5% ethanolic solution of each essential oil at 26 ± 0.1 °C, in a Dichrom P-2000 digital polarimeter equipped with a 100 mm cell at 589 nm. For the determination of relative density, 0.100 mL of each sample was weighed on an analytical balance (Shimadzu AUY220, Kyoto, Japan) at 20 °C. The relative density was calculated in relation to the density of water, which was experimentally determined under the same conditions.

### 4.5. GC-FID and GC–MS Analyses

Samples were prepared by diluting the essential oils and the internal standard, methyl octanoate, in absolute ethanol at final concentrations of 10 and 1 mg/mL, respectively. Subsequently, 1 μL of each sample was injected into a gas chromatograph (5890 Series II, Hewlett-Packard, Palo Alto, CA, USA) equipped with a flame ionization detector (GC-FID), operating in split mode (1:20). Compound separation was performed using a fused silica capillary column with a stationary phase composed of 5% phenyl and 95% dimethylpolysiloxane (30 m × 0.25 mm × 0.25 μm i.d.). The carrier gas flow rate and the temperature program for the column, injector, and detector followed the conditions previously described [53]. The same sample and injection volume (1 μL) were analyzed using a gas chromatograph coupled to a mass spectrometer (GC–MS, model QP-2010 Plus, Shimadzu, Kyoto, Japan). The temperature programs for the column, injector, and interface were identical to those described above [53]. Mass spectra were acquired using a quadrupole detector operating at 70 eV, with a scan range of 40–400 m/z and a scan rate of 0.5 scan/s. Quantification of the compounds present in the essential oils was performed by internal normalization of the chromatographic peak areas obtained by GC-FID, relative to the area of methyl octanoate (internal standard), and the values were expressed as percentages. Compound identification was carried out by GC–MS through the determination of linear retention indices (LRIs), calculated from a homologous series of n-alkanes (C7–C30) injected under the same analytical conditions as the samples [57], and by comparison of the mass spectra with the NIST (2023) database using the GC–MS Solution v.2.53 software (Shimadzu) and reference literature [58].

### 4.6. Chemotype Characterization

To standardize the nomenclature of chemotypes (CTs), the classification key proposed by de Medeiros et al. [53] was adopted. This system is based on the dominance of up to three major compounds in each essential oil sample, so that the chemotype is defined considering one (A), two (A/B), or three (A/B/C) dominant compounds relative to the others.

### 4.7. Calculation of Diversity Indices

The chemical diversity of *B. dracunculifolia* plants in the study region was evaluated based on the mean proportions (%) of essential oil constituents among the accessions. Four diversity indices were applied for this analysis: Shannon–Wiener (H′), Simpson (1 − D), Pielou (J), and Margalef (Mg), as described by Magurran [59]. Calculations were performed in the Python 3.9 environment using the NumPy and Pandas libraries. The relative proportions (pi) of each compound were not subjected to any transformation. The equations used were those of Shannon (1), Simpson (2), Pielou (3), and Margalef (4):

(1) H′ = −Σ pi × ln pi,

(2) 1 − D = 1 − Σ pi^2^,

(3) J = H′/ln S,

(4) Mg = (S − 1)/ln N,

where pi corresponds to the relative proportion of compound *i*, *S* to the number of compounds present, and *N* to the sum of abundances. The interpretation of the indices followed previously published criteria [59,60].

### 4.8. Statistical Analyses

#### 4.8.1. Descriptive and Inferential Statistics

Minimum, maximum, mean values, and relative frequencies of compounds in the essential oils were organized and presented using column and box-plot charts. Analysis of variance (ANOVA) and Tukey’s test (5%) were applied to assess variables according to the sex of the plants sampled. Statistical analyses, as well as the construction of tables and graphs, were performed using Microsoft Excel and GraphPad Prism 9. Geospatial analyses were carried out in the Python 3.9 environment with the support of the NumPy (v1.23.5) [61], Pandas (v1.5.3) [62], Matplotlib(v3.6.2) [63], Seaborn (v0.12.2) [64] and SciPy (v1.10.0) [65] libraries. Georeferenced symbol maps with graduated color scales were generated using the *Seaborn kdeplot* method, and spatial interpolation was conducted using grid data from SciPy [66,67]. Part of the data analysis and figure generation was supported by the artificial intelligence tool ChatGPT-5 (OpenAI, San Francisco, CA, USA), particularly for the development and refinement of Python scripts used for spatial visualization and interpolation [68].

#### 4.8.2. Multivariate Analysis

A data matrix was constructed comprising 50 essential oil samples (independent variables) collected in the state of Rio de Janeiro and 23 compounds (dependent variables). The dependent variables included in the matrix followed the criteria of having a relative proportion equal to or greater than 5% in at least one essential oil sample, or a frequency higher than 70%. This selection considered the relative abundance and recurrence of each constituent, since compounds occurring at low concentrations and low frequency can introduce excessive noise into the data matrix, thereby reducing the interpretative accuracy of multivariate analyses. The inclusion of the most representative compounds allows a more consistent evaluation of the chemical diversity within the species. The data matrix was subjected to clustering using the UPGMA method and the Pearson correlation coefficient as a distance metric for dendrogram construction. The quality of fit between the phenetic and cophenetic matrices was evaluated using the cophenetic correlation coefficient, as described by [69]. The optimal number of groups was defined by considering a minimum similarity of 50% among samples. Additionally, a principal component analysis (PCA) was performed, and a biplot was constructed to represent the scores and factor loadings of the analyzed variables. Hierarchical clustering and PCA were carried out using the software Origin v.2022b (OriginLab Corporation, Northampton, MA, USA). Differences in the overall chemical composition of essential oils were evaluated using a non-parametric, permutation-based test (PERMANOVA) that assesses differences among group centroids in multivariate space without assuming normality. The analysis was based on a Bray–Curtis dissimilarity matrix constructed from the relative proportions (%) of compounds. Three independent factors were tested: Sex (female, male, and sex not reported), Collection period (2023 and 2025) and altitude (0–100 m; 100–400 m and 400–700 m). Analyses were performed in PAST 5.3 (Natural History Museum, University of Oslo, Oslo, Norway).

## 5. Conclusions

The study on *Baccharis dracunculifolia* conducted in the state of Rio de Janeiro revealed a moderate-to-low level of intraspecific chemical diversity. The essential oil yield ranged from 0.34% to 2.17%, relative density from 0.89 to 0.96 g/cm^3^, refractive index from 1.485 to 1.497 nD, and specific optical rotation from −12.56° to +6.80°.

A total of 62 compounds were identified, with *E*-nerolidol standing out as the chemical marker of the species, being detected in all 50 essential oil samples. Multivariate analysis identified five chemical profiles, mainly grouped according to the relative proportions of *E*-nerolidol, spathulenol, germacrene D, bicyclogermacrene, and *E*-caryophyllene, indicating a pattern of moderate-to-low chemical variability among samples.

The chemotypes *E*-nerolidol, *E*-nerolidol/spathulenol, *E*-nerolidol/spathulenol/bicyclogermacrene, and mixed were characterized through the application of a chemical dominance key. Comparison between sexes did not reveal significant differences in the essential oil composition.

It is important to highlight that a slight variation in the chemical composition of *B. dracunculifolia* essential oils was observed between the two collection periods (2023 and 2025), as well as a relationship between the altitude of sampling sites and the chemical profile. Despite these differences, the variations were moderate, with fluctuations mainly in the levels of nerolidol, spathulenol, and bicyclogermacrene. Overall, these fluctuations did not represent substantial alterations in the chemical profile of the species.

This finding indicates that both male and female individuals can be equally used as sources of raw material, simplifying field collection, management, and product standardization under natural or cultivated conditions. Moreover, only slight variations were observed between the two collection periods and among sampling sites, suggesting that the essential oil composition is relatively stable. This stability reinforces the hypothesis that the biosynthetic pathways responsible for terpene production are genetically conserved within the species.

However, an environmental effect related to altitude was detected, emphasizing the importance of studies conducted under controlled conditions, where the environmental influence on the phenotype can be isolated. This approach is crucial for plant breeding programs, as it allows the identification of genotypes that are more responsive to environmental variation and those exhibiting greater phenotypic stability.

Finally, this study provides valuable insights and baseline information for the study, conservation, and sustainable use of native aromatic plants producing essential oils.

## Figures and Tables

**Table 1:** Plant and herbarium accession numbers, collection sites, and geographic coordinates of *Baccharis dracunculifolia* plants collected in Rio de Janeiro, Brazil.

Accession ^1^	Region	City	Longitude (W)	Latitude (S)	Altitude (m)	Herbarium ^2^
BD-P01	Metropolitana	Seropédica	43°41′26.65″	22°42′50.9″	26	RBR 59764
BD-P02	Metropolitana	Japeri	43°36′33.05″	22°39′12.2″	60	RBR 59765
BD-P03	Metropolitana	Japeri	43°34′5.87″	22°39′48.47″	29	RBR 59766
BD-P04	Metropolitana	Nova Iguaçu	43°25′48.38″	22°40′58.72″	38	RBR 59767
BD-P05	Metropolitana	Duque de Caxias	43°22′38.74″	22°40′10.22″	0	RBR 59768
BD-P06	Metropolitana	Magé	43°9′53.74″	22°39′7.08″	19	RBR 59769
BD-P07	Metropolitana	Magé	43°4′49.33″	22°39′28.6″	14	RBR 59770
BD-P08	Metropolitana	Guapimirim	42°57′55.05″	22°40′54.88″	0	RBR 59771
BD-P09	Metropolitana	Rio Bonito	42°39′33.95″	22°42′24.3″	52	RBR 59772
BD-P10	Metropolitana	Rio Bonito	42°37′31.71″	22°43′8.25″	79	RBR 59773
BD-P11	Metropolitana	Rio Bonito	42°34′29.57″	22°46′21.65″	97	RBR 59774
BD-P12	Metropolitana	Rio Bonito	42°29′35.31″	22°48′5.2″	67	RBR 59775
BD-P13	Baixadas Litorâneas	Araruama	42°20′12.17″	22°48′42.72″	35	RBR 59776
BD-P14	Baixadas Litorâneas	Iguaba Grande	42°11′44.61″	22°49′6.83″	41	RBR 59777
BD-P15	Baixadas Litorâneas	Cabo Frio	41°59′52.23″	22°43′16.55″	21	RBR 59778
BD-P16	Baixadas Litorâneas	Búzios	41°53′36.32″	22°46′1.12″	25	RBR 59779
BD-P17	Metropolitana	Itaguaí	43°51′30.0″	22°53′51.94″	17	n/a ^3^
BD-P18	Costa Verde	Mangaratiba	43°54′15.84″	22°55′7.98″	43	RBR 59780
BD-P19	Costa Verde	Mangaratiba	43°59′53.62″	22°56′9.08″	3	RBR 59781
BD-P20	Costa Verde	Mangaratiba	44°6′36.77″	23°1′3.22″	49	n/a
BD-P21	Costa Verde	Angra dos Reis	44°12′51.84″	23°0′33.55″	80	RBR 59782
BD-P22	Costa Verde	Angra dos Reis	44°18′6.57″	22°59′36.95″	60	RBR 59783
BD-P23	Costa Verde	Angra dos Reis	44°26′9.21″	22°57′4.12″	16	RBR 59784
BD-P24	Costa Verde	Angra dos Reis	44°26′29.38″	22°59′52.96″	100	n/a
BD-P25	Costa Verde	Paraty	44°39′8.21″	23°2′43.12″	12	RBR 59785
BD-P26	Costa Verde	Paraty	44°40′42.45″	23°3′21.97″	4	RBR 59786
BD-P27	Costa Verde	Paraty	44°43′25.68″	23°21′9.16″	3	n/a
BD-P28	Metropolitana	Petrópolis	43°07′40″	22°21′54″	693	RBR 62254
BD-P29	Centro-Sul Fluminense	Três Rios	43°09′47″	22°11′23″	310	RBR 62255
BD-P30	Centro-Sul Fluminense	Três Rios	43°06′27″	22°06′34″	342	RBR 62256
BD-P31	Centro-Sul Fluminense	Três Rios	43°01′56″	22°04′5″	258	RBR 62257
BD-P32	Centro-Sul Fluminense	Sapucaia	42°49′24″	21°56′29″	174	RBR 62258
BD-P33	Serrana	Carmo	42°32′59″	21°52′0″	360	RBR 62259
BD-P34	Serrana	Cantagalo	42°25′33″	21°52′10″	481	RBR 62260
BD-P35	Serrana	Cantagalo	42°21′32″	21°56′21″	456	RBR 62261
BD-P36	Serrana	Cantagalo	42°12′17″	21°47′47″	118	RBR 62262
BD-P37	Metropolitana	Japeri	43°39′33″	22°37′23″	39	RBR 62263
BD-P38	Centro-Sul Fluminense	Miguel Pereira	43°31′4″	22°28′29″	674	RBR 62264
BD-P39	Centro-Sul Fluminense	Engo. P. Frontin	43°36′40″	22°29′15″	549	RBR 62265
BD-P40	Médio Paraíba	Valença	43°41′9″	22°20′12″	404	RBR 62266
BD-P41	Médio Paraíba	Valença	43°42′34″	22°17′45″	509	RBR 62267
BD-P42	Médio Paraíba	Valença	43°43′49″	22°15′43″	538	RBR 62268
BD-P43	Médio Paraíba	Valença	43°47′38″	22°17′57″	636	RBR 62269
BD-P44	Médio Paraíba	Valença	43°53′45″	22°16′55″	521	RBR 62270
BD-P45	Médio Paraíba	Valença	43°55′36″	22°18′22″	530	RBR 62271
BD-P46	Médio Paraíba	Barra do Piraí	43°52′17″	22°23′58″	691	RBR 62272
BD-P47	Médio Paraíba	Barra do Piraí	43°55′38″	22°28′35″	407	RBR 62273
BD-P48	Médio Paraíba	Volta Redonda	44°03′1″	22°32′3″	457	RBR 62274
BD-P49	Médio Paraíba	Piraí	43°54′18″	22°38′1″	373	RBR 62275
BD-P50	Metropolitana	Paracambi	43°47′14″	22°41′30″	47	RBR 62276

^1^ Plant accession number; ^2^ Herbarium accession number; ^3^ Accession number not assigned.

**Table 2:** Descriptive statistics of the chemical composition of essential oils from the aerial parts of *Baccharis dracunculifolia*, based on GC-FID and GC–MS analyses.

COMPOUNDS	Type	RT	LRIE	LRIL	Min.	Max.	Mean	Med.	F (%)
*α*-Pinene	MH	8.631	934	932	0.4	2.7	1.3	1.1	74
*β*-Pinene	MH	10.490	975	974	0.5	7.5	1.8	1.3	98
Limonene	MH	12.966	1028	1024	0.9	10.5	4.4	4.2	98
*β*-Elemene	SH	30.218	1393	1389	0.4	1.4	0.8	0.8	90
*E*-Caryophyllene	SH	31.274	1421	1417	1.3	10.1	4.6	4.2	100
Aromadendrene	OS	31.992	1442	1439	0.4	3.6	1.5	1.5	100
*α*-Humulene	SH	32.517	1457	1452	0.7	2.0	1.4	1.5	100
Cabreuva oxide B	OS	32.780	1464	1462	0.7	3.4	2.0	2.0	96
*γ*-Muurolene + Cabreuva oxide D	OC	33.349	1480	1478(79)	0.6	2.3	1.5	1.5	82
Germacrene D	SH	33.485	1484	1480	2.5	15.2	6.6	6.4	100
Bicyclogermacrene	SH	34.015	1499	1500	3.0	14.5	8.9	8.8	100
*α*-Muurolene	SH	34.136	1503	1500	0.4	1.5	0.7	0.7	86
Unidentified Compounds-1510	UC	34.360	1511	-	0.4	1.2	0.8	0.8	92
*γ*-Cadinene	SH	34.562	1518	1513	0.5	1.5	0.9	1.0	94
*δ*-Cadinene	SH	34.867	1528	1522	1.6	5.3	3.4	3.4	100
Unidentified Compounds-1539	UC	35.203	1539	-	1.2	4.1	2.3	2.3	94
Unidentified Compounds-1558	UC	35.771	1559	-	0.6	2.6	1.2	1.1	94
*E*-Nerolidol	OS	36.080	1569	1561	16.8	51.0	29.7	28.3	100
Spathulenol	OS	36.513	1584	1577	3.3	19.5	8.9	8.3	100
Globulol	OS	36.683	1589	1590	1.4	7.0	4.0	4.1	100
Viridiflorol + Cubeban-11-ol	OS	36.914	1597	1592(95)	0.9	3.9	2.1	2.0	74
*α*-Cadinol	OS	38.587	1662	1652	1.4	5.1	2.8	2.8	100
Germacra-4(15),5,10(14)-trien-1-*α*-ol	OS	39.402	1693	1685	0.6	2.9	1.3	1.2	90

Type—Compound class; RT—retention time; LRIE—experimental linear retention index; LRIL—literature linear retention index; f(%)—frequency.

**Table 3:** Characterization of chemotypes based on the dominance criteria of major compounds present in the essential oils of *Baccharis dracunculifolia* from plants collected in situ.

Chemotypes	Type	ID ^1^ (% Compound)
*E*-Nerolidol	A	BD-P01 (31%), BD-P03 (26%), BD-P04 (48%), BD-P07 (28%), BD-P09 (31%), BD-P10 (26%), BD-P13 (41%), BD-P14 (27%), BD-P15 (27%), BD-P17 (28%), BD-P18 (41%), BD-P19 (33%), BD-P22 (25%), BD-P24 (27%), BD-P25 (27%), BD-P26 (30%), BD-P28 (30%), BD-P30 (25%), BD-P31 (32%), BD-P32 (51%), BD-P36 (30%), BD-P37 (28%), BD-P38 (46%), BD-P40 (38%), BD-P42 (40%), BD-P43 (36%), BD-P44 (32%), BD-P45 (35%), BD-P46 (29%), BD-P47 (30%), BD-P48 (29%), BD-P49 (36%), BD-P50 (39%)
*E*-Nerolidol/Spathulenol	A/B	BD-P33 (25%/14%), BD-P34 (32%/19%)
*E*-Nerolidol/Spathulenol/Bicyclogermacrene	A/B/C	BD-P20 (17%/14%/13%)
Mixed	M	BD-P02, BD-P05, BD-P06, BD-P08, BD-P12, BD-P16, BD-P21, BD-P23, BD-P27, BD-P29, BD-P35, BD-P39, BD-P41

^1^ ID referring to Table 1.

**Table 4:** Independent PERMANOVA analyses (Bray–Curtis dissimilarities) assessing the effects of plant sex, collection period and altitude on the essential oil composition of *Baccharis dracunculifolia.*

Analysis (Factor/Levels)	N	*df* (n, d)	SS	MS	*F*	*R* ^2^	*p*-Value
Altitude ^2^ (0–100, 100–400, and 400–700 m)	50	2, 47	0.0941	0.0470	2.296	0.092	0.016
Year ^1^ (2023 × 2025)	50	1, 48	0.0713	0.0713	3.531	0.068	0.005
Sex ^3^ (F × M × NR)	50	2, 47	0.0750	0.0375	1.826	0.059	0.065
Sex (F × M)	26	1, 24	0.0316	0.0316	1.516	0.072	0.199

^1^ Collection period (2023 and 2025) as independent factors. ^2^ Three altitudinal ranges above sea level (m); ^3^ Sex (female, male, sex not reported—NR) and *df*—degrees of freedom; SS—sum of squares; MS—mean square; *R*^2^—proportion of variance (or dissimilarity) explained by the factor; *F*—pseudo-*F* value; *p*-value—probability value obtained from 999 permutations; not significant (*p* > 0.05).

**Table 5:** One-way ANOVA assessing the effect of plant sex (female vs. male) on the relative abundance of the main essential oil compounds of *Baccharis dracunculifolia.*

Compound ^1^	Source of Variation	*df*	SS	MS	F	*p*-Value
*E*-Nerolidol	Treatment (F × M) ^2^	1	0.085	0.085	0.002	0.969
	Residual	24	1.332.026	55.501		
Spathulenol	Treatment (F × M)	1	10.778	10.778	0.944	0.341
	Residual	24	273.973	11.416		
Germacrene D	Treatment (F × M)	1	27.153	27.153	2.505	0.126
	Residual	24	260.122	10.838		
Bicyclogermacrene	Treatment (F × M)	1	31.314	31.314	3.003	0.096
	Residual	24	250.262	10.428		
*E*-Caryophyllene	Treatment (F × M)	1	1.556	1.556	0.471	0.499
	Residual	24	79.241	3.302		
Globulol	Treatment (F × M)	1	0.973	0.973	0.871	0.360
	Residual	24	26.812	1.117		

^1^ Essential oil compounds as independent factor. ^2^ Sex (female and male). Not significant (*p* > 0.05).

## Data Availability

The raw data supporting the conclusions of this article will be made available by the authors upon request.

## Supplementary Materials

The following supporting information can be downloaded at: https://www.mdpi.com/article/10.3390/plants14223443/s1, Table S1: Chemical composition of essential oils from the aerial parts of Baccharis dracunculifolia, based on GC-FID and GC–MS analyses.

## Abbreviations

The following abbreviations are used in this manuscript:

ANOVA	Analysis of Variance
CAM	Multiuser Analytical Center
CT	Chemotype
FID	Flame Ionization Detector
GC-FID	Gas Chromatography with Flame Ionization Detection
GC-MS	Gas Chromatography–Mass Spectrometry
ICMBio	Chico Mendes Institute for Biodiversity Conservation
INEA	State Institute for the Environment (Rio de Janeiro, Brazil)
ISO	International Organization for Standardization
LRI	Linear Retention Index
MH	Monoterpene Hydrocarbons
OM	Oxygenated Monoterpenes
OS	Oxygenated Sesquiterpenes
PCA	Principal Component Analysis
SciPy	Scientific Python Library
SH	Sesquiterpene Hydrocarbons
SisGen	National System for the Management of Genetic Heritage and Associated Traditional Knowledge, Brazil
UPGMA	Unweighted Pair Group Method with Arithmetic Mean
